# Integrated Assessment of Metal-Related Toxicity in a Sentinel Marine Plant, *Posidonia oceanica*, Under Realistic Multi-Element Exposure

**DOI:** 10.3390/ijms27093946

**Published:** 2026-04-29

**Authors:** Paolo Cocci, Martina Fattobene, Raffaele Emanuele Russo, Mario Berrettoni, Francesco Alessandro Palermo

**Affiliations:** 1 School of Biosciences and Veterinary Medicine, University of Camerino, via Gentile III da Varano, 62032 Camerino, Italy; paolo.cocci@unicam.it; 2 Chemistry Division, School of Science and Technology, University of Camerino, via Madonna delle Carceri, 62032 Camerino, Italy; martina.fattobene@unicam.it (M.F.); raffaele.russo@unicam.it (R.E.R.); mario.berrettoni@unicam.it (M.B.)

**Keywords:** *Posidonia oceanica*, trace elements, metal-related toxicity, oxidative stress response

## Abstract

Mediterranean meadows of *Posidonia oceanica* are chronically exposed to complex mixtures of environmental contaminants, including metals and trace elements derived from coastal urbanization, maritime traffic, and industrial activities. This study aimed to assess metal-related toxicity in *P. oceanica* by integrating multi-element burden analysis with a panel of oxidative stress biomarkers. Concentrations of a wide suite of elements were quantified in samples of internal (juvenile), intermediate, and external (adult) leaves, reflecting the ontogenetic structure of the plant. Oxidative responses were evaluated using five biomarkers [i.e., hydrogen peroxide (H_2_O_2_), lipid peroxidation (TBARS), superoxide dismutase (SOD), glutathione S-transferase (GST), and catalase (CAT)] measured on each leaf compartment. Biomarker data were standardized and integrated into a merged Stress Index summarizing overall physiological toxicity. Associations between individual elements, the sum of all measured elements (ΣallElements), the Stress Index, and single biomarkers were explored using Pearson correlation analysis. Juvenile leaves exhibited the highest Stress Index values, elevated H_2_O_2_ and TBARS, and marked activation of SOD and GST, indicating early oxidative toxicity. Intermediate leaves showed a trend toward increased CAT activity, not reaching statistical significance, along with minimal damage, suggesting effective detoxification, whereas adult leaves accumulated higher levels of Fe, Ni, and Pb, but displayed moderate stress responses. Overall, leaf-class structure strongly modulated both exposure and toxicological response. The integration of ΣAllElements with multi-biomarker indices provides a robust framework for diagnosing metal-related toxicity in *P. oceanica* under realistic multi-element exposure scenarios.

## 1. Introduction

*Posidonia oceanica* seagrass meadows represent a key ecological component of Mediterranean coastal ecosystems, contributing to sediment stabilization, water quality improvement, biodiversity support, and long-term carbon sequestration [1,2]. However, these habitats are increasingly threatened by anthropogenic pressures such as coastal urbanization, maritime activities, desalination effluents, and contaminant inputs [3]. Among these stressors, heavy metals and trace elements are of particular concern due to their persistence and tendency to accumulate in sediments and plant tissues [4,5,6]. Previous studies have shown that *P. oceanica* can uptake and compartmentalize trace elements across different tissues, with concentrations often reflecting environmental levels in seawater and sediments [6]. Moreover, metal exposure has been associated with alterations in physiological processes, including photosynthesis and oxidative balance, as well as with the activation of detoxification and antioxidant mechanisms [4]. Recent investigations have further highlighted the complexity of stress responses in natural environments, where multiple stressors—including trace elements, temperature, and nutrient variability—interact and influence plant health and resilience [5].

At the cellular level, metal-induced stress is largely mediated by the overproduction of reactive oxygen species (ROS), leading to oxidative damage such as lipid peroxidation and DNA impairment [7,8,9]. In response, seagrasses activate enzymatic and non-enzymatic antioxidant systems, including superoxide dismutase (SOD), catalase (CAT), glutathione, and related pathways [10,11,12]. However, most studies have focused on single contaminants or isolated biomarkers, limiting the ecological relevance of the results. In natural conditions, organisms are exposed to complex mixtures of elements, whose combined effects may be additive or interactive. Therefore, cumulative indices such as the sum of all detected elements (ΣAllElements) provide a more realistic representation of environmental exposure and its biological consequences. Additionally, *P. oceanica* shoots exhibit a clear leaf-age gradient (juvenile, intermediate, and adult leaves), which influences both contaminant accumulation and physiological responses [6,13].

In this context, the present study aims to quantify trace element distribution across different leaf compartments (juvenile, intermediate, and adult), to assess oxidative stress biomarkers (H_2_O_2_, TBARS, SOD, CAT, GST), and to integrate these parameters into a composite stress index, in order to better elucidate compartment-specific responses and the overall ecotoxicological impact of mixed metal exposure.

## 2. Results and Discussion

The concentrations of contaminants in *P. oceanica* leaves differed markedly among juvenile, intermediate, and adult leaf portions (Table 1), highlighting a sort of specificity in contaminant accumulation.

The ΣAllElements was highest in juvenile leaves (1521.43 ppm), followed by adult leaves (987.31 ppm), and was lowest in intermediate leaves (902.97 ppm). Several elements exhibited clear leaf-class-specific patterns. Both Al and Cr showed their maximum concentration in juvenile leaves, decreasing sharply in intermediate and adult leaves. A similar trend was observed for Mo, which was highest in juvenile leaves and declined in intermediate and adult leaves. In contrast, Fe, Ni, Co, and Mn progressively increased toward older tissues. In particular, Mn showed the most pronounced increase, more than doubling from juvenile (91.2 ppm) to adult leaves (211.8 ppm). A different pattern was observed for Pb, which was detected exclusively in adult leaves, indicating preferential accumulation of this non-essential and potentially toxic element in the oldest and most exposed leaf tissues. Cu and Zn displayed a distinct pattern, with maximum concentrations in intermediate leaves (Cu: 30.6 ppm; Zn: 104.4 ppm), compared with juvenile (Cu: 28.4 ppm; Zn: 92.5 ppm) and adult leaves (Cu: 19.7 ppm; Zn: 96.1 ppm). This suggests active regulation or redistribution rather than simple surface accumulation. Sr also increased markedly toward adult leaves, reaching 224.1 ppm compared with 96.1 ppm in juvenile leaves. Elements such as As, Ba, V, and Ti showed relatively limited variation among leaf classes, whereas Ag peaked in intermediate leaves and remained low in juvenile and adult tissues. Several elements (Be, Bi, Sb, Tl) were below detection limits in all leaf portions. Overall, these results demonstrate that juvenile leaves concentrate the highest total elemental load, largely driven by elevated Al and Mo, whereas adult leaves act as sinks for transition metals and toxic elements such as Fe, Mn, Ni, and Pb. Intermediate leaves display selective enrichment of Cu and Zn. This heterogeneous distribution of elements among leaf classes highlights the importance of considering leaf position within the shoot when assessing contaminant exposure in *P. oceanica* [6].

**Figure 1:**
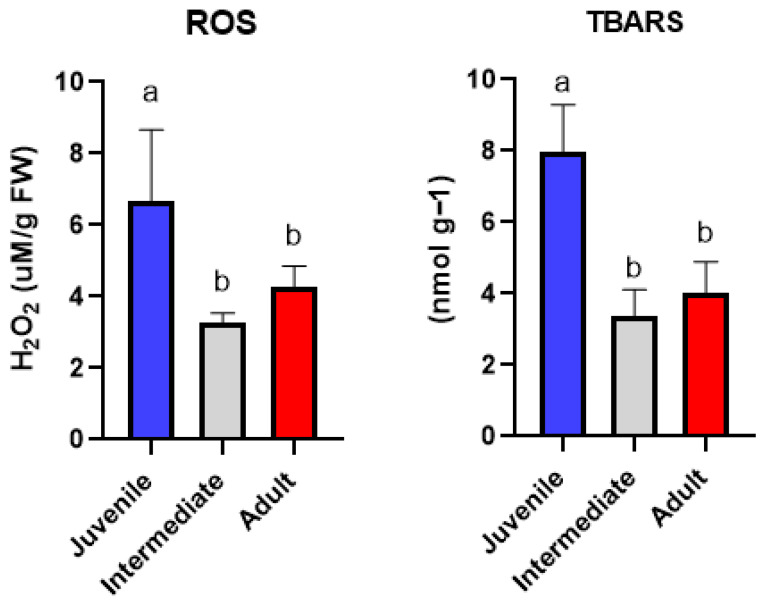
Hydrogen peroxide (H_2_O_2_) concentration and lipid peroxidation (TBARS) in *P. oceanica* leaves according to leaf class (juvenile, intermediate, adult). Data are shown as mean ± SD. Different letters indicate statistically significant differences among groups (*p* < 0.05).

The analysis of oxidative stress biomarkers revealed a clear and comprehensible differentiation among leaf classes, highlighting a strong modulation of redox status. Hydrogen peroxide concentrations differed significantly among leaf classes, with juvenile leaves exhibiting the highest values, followed by adult and intermediate leaves (Figure 1). The significantly elevated H_2_O_2_ concentrations detected in juvenile leaves reflect enhanced oxidative pressure in younger tissues of *P. oceanica*. The accumulation of metals in these tissues, therefore, likely represents a physiological consequence of oxidative damage. As older tissues, external leaves are subject to prolonged exposure to environmental stress and metal accumulation, yet the absence of extreme H_2_O_2_ levels suggests successful long-term acclimation and redox stabilization [14]. Lipid peroxidation, expressed as TBARS, also showed highly significant differences among leaf classes (Figure 1). Juvenile leaves displayed the highest TBARS levels, indicating enhanced oxidative membrane damage, whereas intermediate and adult leaves showed the lowest values, consistent with effective ROS control. The significantly elevated TBARS levels observed in juvenile leaves indicate enhanced lipid peroxidation in younger tissues of *P. oceanica*, reflecting a higher degree of oxidative stress. Lipid peroxidation is a downstream consequence of excessive reactive oxygen species accumulation, and its increase in juvenile leaves is consistent with the elevated H_2_O_2_ concentrations detected in the same leaf class [15]. The low TBARS concentrations measured in intermediate and adult leaves suggest effective control of oxidative damage, despite exposure to environmental stressors and metal accumulation, suggesting that older tissues experience sustained but moderated oxidative damage, potentially reflecting long-term acclimation mechanisms rather than acute oxidative stress.

**Figure 2:**
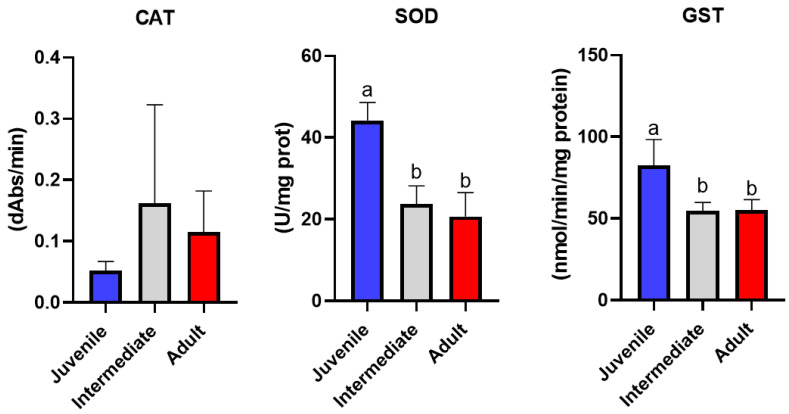
Catalase (CAT), superoxide dismutase (SOD) and glutathione-S transferase (GST) activity in *P. oceanica* leaves according to leaf class (juvenile, intermediate, adult). Data are shown as mean ± SD. Different letters indicate statistically significant differences among groups (*p* < 0.05).

While hydrogen peroxide accumulation and lipid peroxidation provide direct evidence of oxidative pressure and membrane damage, the extent to which *P. oceanica* leaves can respond to these processes depends on the activation of enzymatic antioxidant defenses [4,10]. To clarify whether corresponding detoxification responses accompanied the observed ROS patterns, the activities of key antioxidant and phase II enzymes were examined across the different leaf classes (Figure 2). Catalase (CAT) activity did not differ significantly among leaf classes, despite a tendency toward higher mean values in intermediate and adult leaves. The absence of statistical significance, combined with high intra-class variability, suggests that CAT activity is not strictly regulated by leaf position but rather reflects heterogeneous detoxification responses among individual shoots.

Superoxide dismutase (SOD) activity differed significantly among leaf classes, indicating a clear morphological pattern (Figure 2). Juvenile leaves exhibited the highest SOD activity, whereas intermediate and adult leaves showed substantially lower values. The significantly elevated SOD activity observed in juvenile leaves highlights the central role of this enzyme in the early antioxidant defense of *P. oceanica* [16]. As the first enzymatic barrier against reactive oxygen species, SOD catalyzes the dismutation of superoxide radicals into hydrogen peroxide, and its enhanced activity in juvenile tissues is consistent with the higher metabolic rates and increased ROS production associated with active growth. The sharp contrast between juvenile and older leaf portions suggests that SOD-mediated detoxification is particularly important in younger leaves, where oxidative pressure is greatest. This interpretation is further supported by the concomitant increase in hydrogen peroxide concentrations measured in juvenile leaves, indicating that enhanced SOD activity contributes to elevated downstream ROS pools rather than complete detoxification. In contrast, intermediate and adult leaves exhibited significantly lower and statistically indistinguishable SOD activities. This pattern suggests a shift in antioxidant strategy with leaf ontogeny, whereby older tissues rely less on superoxide scavenging and more on alternative detoxification pathways or structural tolerance mechanisms [17]. Notably, the strong statistical signal detected for SOD contrasts with the absence of significant differences observed for catalase activity. This decoupling among antioxidant enzymes underscores the importance of using multi-biomarker panels, as single enzymes may respond selectively depending on tissue age and physiological status. In this context, SOD potentially emerges as a highly sensitive and discriminant biomarker for assessing oxidative stress gradients within *P. oceanica* shoots. Glutathione S-transferase (GST) activity differed significantly among leaf classes (Figure 2). Juvenile leaves showed the highest GST activity, whereas intermediate and adult leaves exhibited markedly lower and comparable values. The significantly higher GST activity observed in juvenile leaves highlights the importance of glutathione-dependent detoxification pathways in younger *P. oceanica* tissues. GST enzymes play a key role in phase II detoxification by catalyzing the conjugation of reduced glutathione to electrophilic compounds, including lipid peroxidation products and metal-induced reactive intermediates. The enhanced GST activity in juvenile leaves is therefore consistent with the elevated oxidative pressure and higher metabolic activity characterizing these tissues. The close correspondence between GST and SOD activity patterns suggests a coordinated antioxidant response in juvenile leaves, where increased superoxide scavenging is coupled with enhanced detoxification of downstream oxidative by-products [18]. This coupling supports the interpretation that juvenile leaves represent a “stress-active” compartment within the shoot, characterized by rapid ROS turnover and strong activation of enzymatic defense systems [19]. In contrast, intermediate and adult leaves exhibited significantly lower and statistically indistinguishable GST activities, indicating a reduced reliance on glutathione-mediated detoxification in older tissues. This may reflect a shift toward alternative tolerance strategies, such as structural compartmentalization of contaminants or long-term acclimation mechanisms, rather than inducible enzymatic responses. Together with SOD, GST emerges as a sensitive and informative biomarker for detecting sub-lethal metal-related stress in *P. oceanica.*

**Figure 3:**
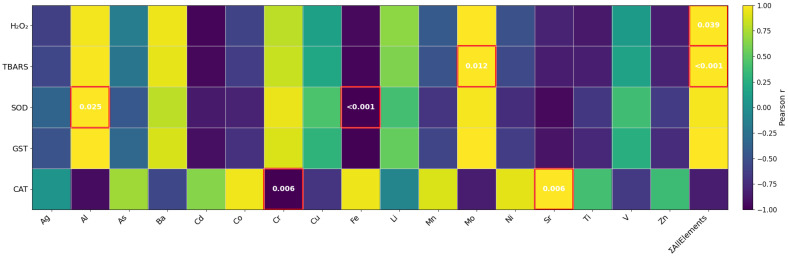
Pearson correlations between elemental concentrations (including ΣAllElements) and individual oxidative stress biomarkers (H_2_O_2_, TBARS, SOD, GST and CAT) across leaf classes of *P. oceanica* (*n* = 3). Color scale represents Pearson correlation coefficients (r). Statistically significant correlations (*p* ≤ 0.05) are highlighted and annotated with *p*-values. All the correlations are shown for exploratory purposes.

Correlation analyses revealed biomarker-specific relationships between elemental concentrations and oxidative stress responses across leaf classes (Figure 3). Hydrogen peroxide (H_2_O_2_) and TBARS showed similar correlation patterns, with positive associations with Al, Ba, Cr, Mo, and ΣAllElements, and negative correlations with Fe, Zn, Sr, Cd and Ti. Significant relationships were observed between H_2_O_2_ and ΣAllElements, and between TBARS and both Mo and ΣAllElements (*p* < 0.05), supporting a link between cumulative element exposure and oxidative damage. The close correspondence between H_2_O_2_ and TBARS supports that ROS accumulation in juvenile leaves is accompanied by enhanced lipid peroxidation. SOD activity followed a comparable trend, with significant correlations (*p* < 0.05) detected with Al (positive) and Fe (negative), indicating a close association between the integrated elemental burden and ROS-related stress gradient across leaf classes. GST showed a similar but less robust pattern compared to other biomarkers, with strong correlations that did not reach statistical significance. In contrast, CAT activity displayed a distinct and partly opposite pattern, with significant correlations (*p* < 0.05) with Cr (negative) and Sr (positive), indicating a more specific and less uniform response of this enzyme. Overall, these results support the role of cumulative multi-element exposure, particularly ΣAllElements, rather than single contaminants, in shaping oxidative stress responses in *P. oceanica*, while highlighting enzyme-specific differences in antioxidant regulation. The recurrent association of Mo, together with Al and ΣAllElements, with multiple biomarkers suggests that elements preferentially accumulated in juvenile leaves are closely linked to enhanced ROS production, antioxidant enzyme activation, and lipid peroxidation. Although some of these elements are essential micronutrients, their enrichment in metabolically active tissues likely increases redox turnover and antioxidant demand, contributing to the “stress-active” phenotype of juvenile leaves. Conversely, elements such as Fe and Sr, which accumulate predominantly in adult leaves, were consistently correlated with most oxidative biomarkers. This indicates that element accumulation in older tissues does not necessarily translate into increased oxidative stress, likely reflecting long-term acclimation, compartmentalization, or structural tolerance mechanisms. The contrasting behavior of CAT further supports the notion that antioxidant responses are enzyme-specific. This pattern is consistent with the absence of significant leaf-class differences in CAT activity and suggests a more constitutive or context-dependent role for this enzyme.

**Figure 4:**
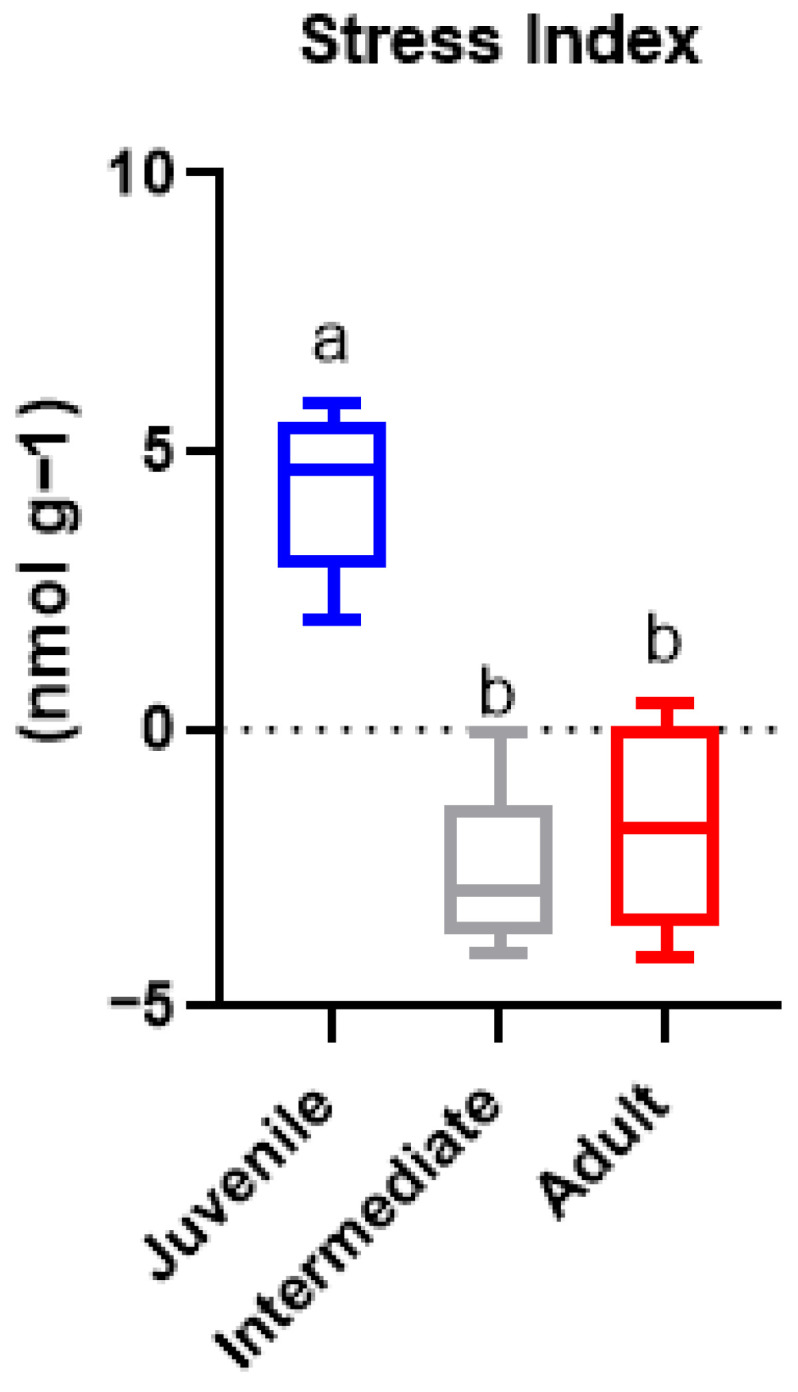
Stress Index (sum of z-scores across H_2_O_2_, TBARS, SOD, GST and CAT) by leaf class (Juvenile, Intermediate, Adult). Boxplots show median, interquartile range and whiskers (*n* = 5 replicates per class). Positive values indicate higher integrated oxidative stress. Different letters indicate statistically significant differences among groups (*p* < 0.05).

The Stress Index, calculated as the sum of standardized (z-score) values of H_2_O_2_, TBARS, SOD, GST and CAT, showed marked differences among leaf classes (Figure 4). Juvenile leaves exhibited consistently positive Stress Index values, with a median around 4–5, indicating a high integrated oxidative stress status. The interquartile range and whiskers revealed moderate variability among replicates, but all internal samples remained well above zero. In contrast, intermediate leaves displayed strongly negative Stress Index values, with a median close to −3, reflecting the lowest overall oxidative stress among the three leaf classes. Variability within this group was limited, and most replicates clustered tightly at negative values, indicating a stable low-stress condition. Adult leaves showed intermediate Stress Index values, predominantly negative but with a wider dispersion than intermediate leaves. The median Stress Index for adult leaves was approximately −2, with some replicates approaching zero or slightly positive values, suggesting moderate and more heterogeneous stress conditions compared to the other classes. Overall, the Stress Index followed a clear gradient among leaf portions, decreasing from juvenile > adult > intermediate leaves. This pattern indicates that juvenile leaves represent the most stress-active compartment within *P. oceanica* shoots, whereas intermediate leaves maintain the lowest integrated oxidative stress, and adult leaves occupy an intermediate position characterized by greater variability among replicates.

**Figure 5:**
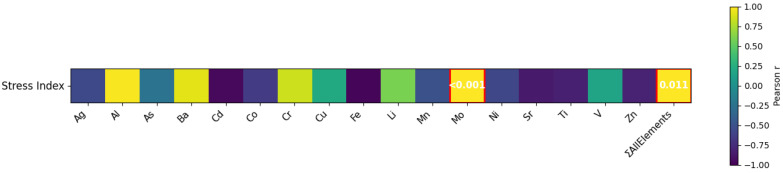
Pearson correlations between element concentrations (including ΣAllElements) and the integrated Stress Index across leaf classes of *P. oceanica* (*n* = 3). Color scale represents Pearson correlation coefficients (r). Statistically significant correlations (*p* ≤ 0.05) are highlighted and annotated with *p*-values. All the correlations are shown for exploratory purposes.

The correlation analysis between elemental concentrations and the integrated Stress Index revealed distinct association patterns across leaf classes (Figure 5). The cumulative elemental load (ΣAllElements) showed a strong positive and statistically significant correlation with the Stress Index (*p* ≤ 0.05), indicating that higher overall elemental loads are associated with increased oxidative stress. Among individual elements, Mo also exhibited a significant positive correlation with the Stress Index (r = 1.00; *p* ≤ 0.05), suggesting a potential role of this element in affecting oxidative responses. Although other elements such as Al (r = 0.98), Ba (r = 0.92), and Cr (r = 0.85) showed positive associations, these relationships did not reach statistical significance.

In contrast, several elements showed strong negative correlations with the Stress Index, including Fe (r = −0.97), Sr (r = −0.86), Ti (r = −0.82), and Zn (r = −0.80). These elements tended to accumulate preferentially in intermediate and adult leaves, which were characterized by lower Stress Index values. However, these trends were not statistically significant and should be interpreted cautiously. Overall, these results support the relevance of cumulative multi-element exposure in determining oxidative stress responses in *P. oceanica*. In particular, the significant positive correlations observed between the Stress Index and both ΣAllElements and Mo highlight the importance of integrated elemental load and specific key elements in driving oxidative stress patterns. Conversely, the negative trends observed for Fe, Sr, Zn, and Ti indicate that accumulation of these elements in older leaf portions does not translate into increased oxidative stress as measured by the integrated index. This pattern likely reflects long-term acclimation and compartmentalization processes in older leaves, which may act as sinks for elements without triggering strong oxidative responses.

## 3. Materials and Methods

### 3.1. Study Area and Sampling

*P.* leaves were randomly collected along the Marine Protected Area of Tremiti Island Archipelago (Adriatic Sea, FG, Italy), a coastal environment characterized by *P. oceanica* meadows exposed to varying degrees of anthropogenic pressure, including maritime traffic and tourism activities. In particular, samples were collected at Cala Matano, a semi-enclosed bay located on the southern coast of San Domino Island, known for its reduced hydrodynamics and higher potential for contaminant accumulation. The shallow sandy seabed at the study site supports well-developed *P. oceanica* meadows. The geomorphological characteristics of Cala Matano, together with its partial confinement, make it a suitable site for investigating metal accumulation and associated biological responses. Sampling was carried out by SCUBA divers at depths ranging approximately between 14 and 17 m. *P. oceanica* shoots were collected manually, and leaves were immediately separated according to their position into internal (juvenile), intermediate, and external (adult) leaves, following established classification criteria. After collection, *P. oceanica* leaves were immediately stored at −20 °C until further analysis, in accordance with established protocols for preserving biochemical and elemental integrity. It should be noted that *P. oceanica* shoots within each sampling site are interconnected by a continuous rhizome network, and therefore all collected samples belong to the same seagrass meadow. Therefore, shoots share comparable environmental conditions and a common physiological background, making them suitable for integrated ecophysiological and ecotoxicological assessments. This sampling assumption is consistent with previous studies on *P. oceanica*, where interconnected shoots within a meadow were considered as part of the same functional unit for evaluating multiple abiotic stresses [19].

### 3.2. Analytical Procedures for Trace Elements Analysis

Prior to the analysis, samples were thawed and carefully cleaned to remove epiphytic algae, debris, and necrotic plant tissues. Approximately 100 mg of oven-dried *P. oceanica* leaf tissue, homogenized using a mortar, were transferred into test tubes and digested using an open-vessel protocol. The acid digestion step was performed by adding 3 mL of ultrapure nitric acid (HNO_3_, 65% *w*/*w*, Suprapur^®^ grade, Merck, Darmstadt, Germany). Each tube was covered with a watch glass and placed in a sand bath maintained at 80 °C for 1 h to allow sample decomposition. After this initial digestion phase, hydrogen peroxide (H_2_O_2_, 30% *w*/*w*, Suprapur^®^ grade; Merck, Darmstadt, Germany) was introduced to convert nitrous acid back into nitric acid, thereby supporting further oxidation of the matrix. The digestion temperature was subsequently increased to 100 °C and maintained for an additional hour. At the end of the procedure, a clear solution corresponding to the extractable fraction and free from residual particulates was obtained and diluted using MilliQ water. The concentration of trace elements (including Ag, Al, As, Ba, Be, Bi, Cd, Co, Cr, Cu, Fe, Li, Mn, Mo, Ni, Pb, Sb, Se, Sr, Ti, Tl, V and Zn) was determined using Inductively Coupled Plasma Optical Emission Spectrometry (ICP-OES). Quantification was performed using external calibration curves over an appropriate concentration range (ppb–ppm). Quality assurance included instrument calibration, procedural blanks, and replicate measurements to ensure analytical accuracy and precision. All reagents were of suprapure or analytical grade, and MilliQ water was used for all dilutions. Glassware and digestion vessels were acid-cleaned prior to use to prevent contamination. Procedural blanks were processed together with samples to monitor potential background contributions. Calibration performance and instrumental stability were verified by analyzing quality control standards during each analytical batch. Analytical precision was evaluated through replicate measurements, and relative standard deviations were maintained within acceptable analytical limits. Samples exceeding the calibration range were appropriately diluted to ensure accurate quantification within the validated calibration interval. Limits of detection (LOD) and quantification (LOQ) were automatically determined by the instrument software from calibration data (Qtegra ISDS Software v. 2.11 FR1, 2021 controlling the iCAP PRO Series ICP-OES; Thermo Fisher Scientific, Waltham, MA, USA). Element concentrations are expressed as ppm (mg kg^−1^ dry weight). Values below the limit of quantification (LOQ) were reported as <LOQ. To assess cumulative exposure, the variable ΣAllElements was calculated as the sum of all quantified elements above detection limits.

### 3.3. Antioxidant Enzyme Analysis

A portion of adult, intermediate, and juvenile leaf blades (0.5 g) was homogenized on ice with a phosphate-buffered solution (0.1 M; pH 7.5). Then, the homogenate was centrifuged at 10,000 rpm for 15 min at 4 °C, and the resulting supernatant was stored at −80 °C for biochemical assays. Total protein concentrations in crude enzyme extract were determined according to the Bradford Protein Assay (Biorad Laboratories, Inc.; Hercules, CA, USA). Commercial kits were used for the determination of the activities of superoxide dismutase (Total Superoxide Dismutase Activity Assay Kit; MyBioSource MBS2540402, MyBioSource, Inc.; San Diego, CA, USA), catalase (Catalase Assay Kit; MyBioSource MBS2540413, MyBioSource, Inc.; San Diego, CA, USA) and glutathione S transferase (Glutathione S-transferase Microplate Assay Kit MBS8243172, MyBioSource, Inc.; San Diego, CA, USA). All procedures and sample dilutions were performed following the protocol described in the respective operating manuals.

### 3.4. Oxidative Stress Indices

We measured the oxidative stress level quantifying the ROS production and the membrane lipid peroxidation (Thiobarbituric acid reactive substances, TBARS). Briefly, Hydrogen peroxide (H_2_O_2_) concentrations were determined using a fluorometric assay (Hydrogen Peroxide Assay Kit; Merck, Darmstadt, Germany; LOD: 0.03 μM) based on peroxidase-mediated oxidation of a red fluorescent substrate. Leaf extracts previously obtained from *P. oceanica* tissues were used for the analysis. Briefly, aliquots of leaf extracts were transferred into wells of a 96-well microplate. For quantification, a standard curve was prepared using hydrogen peroxide standards ranging from 0 to 10 μM. Serial dilutions of the working solution were prepared to generate the calibration standards (10, 3, 1, 0.3, 0.1, 0.03, 0.01 and 0 μM). Fifty microliters of each standard solution were added to the corresponding wells. The reaction was initiated by adding 50 μL of a freshly prepared Master Mix to each well. The Master Mix consisted of red peroxidase substrate (50 μL), peroxidase enzyme solution (20 units mL^−1^; 200 μL), and assay buffer (4.75 mL). Plates were gently mixed and incubated at room temperature for 30 min in the dark to allow development of fluorescence. Fluorescence intensity was measured using a microplate reader at excitation and emission wavelengths of 540 and 590 nm, respectively. All standards and samples were analyzed in duplicate. Background fluorescence corresponding to the blank (0 μM H_2_O_2_) was subtracted from all measurements. Hydrogen peroxide concentrations in the samples were then calculated by interpolation from the standard calibration curve and expressed relative to fresh weight of the leaf material (μmol g^−1^ FW). Lipid peroxidation was quantified by measuring thiobarbituric acid reactive substances (TBARS), using malondialdehyde (MDA) as the main indicator of membrane oxidative damage [20]. A standard calibration curve was prepared using 1,1,3,3-tetraethoxypropane (Merck, Darmstadt, Germany). Briefly, aliquots of *P. oceanica* leaf were homogenized in 0.9% NaCl using an Ultra-Turrax homogenizer (IKA^®^ ULTRA-TURRAX^®^, Merck, Darmstadt, Germany) and incubated for 15 min at 37 °C. Subsequently, a reaction solution containing 0.8 M HCl, 12.5% trichloroacetic acid (TCA), and 1.0% thiobarbituric acid (TBA) was added to the mixture. Samples were then incubated at 100 °C for 10 min. After incubation, samples were centrifuged at 5000 rpm for 15 min at 4 °C, and the supernatant was collected for spectrophotometric analysis. Absorbance was measured at 535 nm using a spectrophotometer (JASCO V-550, JASCO Europe Srl; Cremella, Italy). MDA concentrations were calculated from the standard calibration curve and expressed as nmol TBARS g^−1^ of fresh weight (FW) of leaf tissue. All measurements were performed in duplicate.

### 3.5. Integrated Stress Index Calculation

The integrated stress index was calculated to obtain an integrated and comparable measure of oxidative status across the three leaf classes. For each biomarker quantified, raw values were first checked for distribution and then transformed into standardized z-scores (z = (x − μ)/σ) where x is the biomarker value of one replicate, μ is the mean of that biomarker calculated from the total samples, and σ is the corresponding standard deviation. This normalization removed scale differences among endpoints expressed in heterogeneous units and gave equal weight to H_2_O_2_, TBARS, SOD, GST, and CAT. For each sample, the Stress Index was computed as the arithmetic average of its five z-scores: SI_i_ = (zH_2_O_2i_ + zTBARS_i_ + zSOD_i_ + zGST_i_ + zCAT_i_)/5, with higher positive values indicating greater oxidative imbalance and activation of antioxidant defenses, and negative or near-zero values reflecting low stress conditions.

### 3.6. Statistical Analysis

Data are presented as mean ± standard deviation. Normality of data distribution was assessed using the Shapiro–Wilk test, and homogeneity of variances was verified using Levene’s test. Pearson correlation coefficients (two-tailed) were used to evaluate the relationships between oxidative stress biomarkers, Stress Index, and trace elements concentration in *P. oceanica* leaves. Differences in individual oxidative stress biomarkers (H_2_O_2_, TBARS, SOD, GST, and CAT) among leaf classes (juvenile, intermediate, adult) were analyzed using one-way ANOVA, followed by Tukey’s post hoc test for multiple comparisons. Statistical significance was set at *p* < 0.05. All analyses were performed using GraphPad Prism version 8.

## 4. Conclusions

This study shows that oxidative stress responses in *P. oceanica* are primarily driven by leaf compartmentalization and cumulative elemental load rather than by single contaminants. Juvenile (internal) leaves were identified as the most metabolically active and stress-responsive compartment, while intermediate leaves exhibited the lowest oxidative stress, suggesting effective redox regulation. Adult (external) leaves accumulated higher element concentrations but showed moderate oxidative responses, consistent with acclimation processes. The integration of multiple biomarkers into a composite Stress Index proved effective in capturing coherent physiological patterns and was strongly associated with cumulative elemental exposure (ΣAllElements). Overall, these findings highlight the importance of considering leaf-class structure and support the use of integrated metrics for assessing contaminant-related stress in seagrass ecosystems. From an applied perspective, emerging approaches such as nanozyme-based systems have been proposed to mitigate metal-induced oxidative stress in plants [21]. In addition, recent advances in microfluidic technologies offer promising tools for the detection and removal of heavy metals from environmental matrices, supporting more effective monitoring and remediation strategies [22].

## Figures and Tables

**Table 1:** Concentrations of all detectable elements (ppm) and ΣAllElements across leaf classes of *P. oceanica.* Data are reported as mean ± standard deviation (SD) estimated from replicate measurements. ΣAllElements was calculated as the sum of all quantified elements above detection limits. loq = limit of quantification.

Element	Juvenile Leaf	Intermediate Leaf	Adult Leaf
Ag	0.76 ± 0.07	7.10 ± 0.57	0.46 ± 0.04
Al	996.74 ± 88.42	260.02 ± 22.99	189.13 ± 16.38
As	0.85 ± 0.07	0.81 ± 0.07	1.02 ± 0.09
Ba	10.32 ± 0.88	8.25 ± 0.76	9.28 ± 0.84
Be	<loq	<loq	<loq
Bi	<loq	<loq	<loq
Cd	1.42 ± 0.12	1.45 ± 0.13	1.43 ± 0.12
Co	1.96 ± 0.19	2.45 ± 0.24	3.60 ± 0.32
Cr	4.56 ± 0.44	4.01 ± 0.33	3.57 ± 0.32
Cu	28.36 ± 2.31	30.60 ± 2.71	19.68 ± 1.61
Fe	153.39 ± 12.66	166.61 ± 15.32	168.59 ± 14.25
Li	6.62 ± 0.54	3.70 ± 0.31	6.59 ± 0.57
Mn	91.19 ± 8.15	106.41 ± 8.94	211.76 ± 19.65
Mo	6.64 ± 0.60	3.00 ± 0.27	3.43 ± 0.30
Ni	25.74 ± 2.47	28.63 ± 2.62	40.04 ± 3.33
Pb	<loq	<loq	5.333 ± 0.362
Sb	<loq	<loq	<loq
Se	1.033 ± 0.10	1.087 ± 0.11	<loq
Sr	96.08 ± 9.40	169.88 ± 14.75	224.11 ± 20.47
Ti	1.51 ± 0.13	2.66 ± 0.24	1.89 ± 0.18
Tl	<loq	<loq	<loq
V	1.74 ± 0.16	1.88 ± 0.19	1.29 ± 0.12
Zn	92.52 ± 7.55	104.42 ± 9.12	96.11 ± 9.32
Σ All	1521.43 ± 134.08	902.97 ± 72.73	987.31 ± 83.87

## Data Availability

The original contributions presented in this study are included in the article. Further inquiries can be directed to the corresponding author.
